# Vector competence of populations of *Aedes aegypti* from three distinct cities in Kenya for chikungunya virus

**DOI:** 10.1371/journal.pntd.0005860

**Published:** 2017-08-18

**Authors:** Sheila B. Agha, Edith Chepkorir, Francis Mulwa, Caroline Tigoi, Samwel Arum, Milehna M. Guarido, Peris Ambala, Betty Chelangat, Joel Lutomiah, David P. Tchouassi, Michael J. Turell, Rosemary Sang

**Affiliations:** 1 International Centre of Insect Physiology and Ecology, Nairobi, Kenya; 2 Centre for Viral Zoonosis, Department of Medical Virology, University of Pretoria, Pretoria, South Africa; 3 Institute of Primate Research, Nairobi, Kenya; 4 Arbovirus/Viral Hemorrhagic Fever Laboratory, Center for Virus Research, Kenya Medical Research Institute, Nairobi, Kenya; 5 VectorID LLC, Frederick, Maryland, United States of America; University of California, Davis, UNITED STATES

## Abstract

**Background:**

In April, 2004, chikungunya virus (CHIKV) re-emerged in Kenya and eventually spread to the islands in the Indian Ocean basin, South-East Asia, and the Americas. The virus, which is often associated with high levels of viremia in humans, is mostly transmitted by the urban vector, *Aedes aegypti*. The expansion of CHIKV presents a public health challenge both locally and internationally. In this study, we investigated the ability of *Ae*. *aegypti* mosquitoes from three distinct cities in Kenya; Mombasa (outbreak prone), Kisumu, and Nairobi (no documented outbreak) to transmit CHIKV.

**Methodology/Principal findings:**

*Aedes aegypti* mosquito populations were exposed to different doses of CHIKV (10^5.6–7.5^ plaque-forming units[PFU]/ml) in an infectious blood meal. Transmission was ascertained by collecting and testing saliva samples from individual mosquitoes at 5, 7, 9, and 14 days post exposure. Infection and dissemination were estimated by testing body and legs, respectively, for individual mosquitoes at selected days post exposure. Tissue culture assays were used to determine the presence of infectious viral particles in the body, leg, and saliva samples. The number of days post exposure had no effect on infection, dissemination, or transmission rates, but these rates increased with an increase in exposure dose in all three populations. Although the rates were highest in *Ae*. *aegypti* from Mombasa at titers ≥10^6.9^ PFU/ml, the differences observed were not statistically significant (χ2 ≤ 1.04, DF = 1, P ≥ 0.31). Overall, about 71% of the infected mosquitoes developed a disseminated infection, of which 21% successfully transmitted the virus into a capillary tube, giving an estimated transmission rate of about 10% for mosquitoes that ingested ≥10^6.9^ PFU/ml of CHIKV. All three populations of *Ae*. *aegypti* were infectious as early as 5–7 days post exposure. On average, viral dissemination only occurred when body titers were ≥10^4^ PFU/ml in all populations.

**Conclusions/Significance:**

Populations of *Ae*. *aegypti* from Mombasa, Nairobi, and Kisumu were all competent laboratory vectors of CHIKV. Viremia of the infectious blood meal was an important factor in *Ae*. *aegypti* susceptibility and transmission of CHIKV. In addition to viremia levels, temperature and feeding behavior of *Ae*. *aegypti* may also contribute to the observed disease patterns.

## Author summary

A chikungunya epidemic recently occurred in Mandera, Northern Kenya, with over 1,700 cases reported. The disease epidemics are linked to the urban vector, *Aedes aegypti*. This mosquito species is rapidly expanding its range and it is currently abundant in and around the major urban cities of Kenya. In this study, we demonstrated the ability of *Ae*. *aegypti* from three distinct cities of Kenya to transmit chikungunya virus (CHIKV) under laboratory conditions. Our findings showed that populations of *Ae*. *aegypti* from Mombasa, Kisumu, and Nairobi were competent vectors for CHIKV. Overall, about 60% of the *Ae*. *aegypti* that ingested ≥10^6.9^ plaque-forming units of virus/ml became infected and about 10% of the virus-exposed mosquitoes transmitted virus to a capillary tube. Vector competence remains a prerequisite in disease risk assessment, while surveillance and control of *Ae*. *aegypti* should remain the main focus in many disease control programs and should be performed routinely.

## Introduction

Chikungunya is a re-emerging mosquito-borne infectious disease caused by chikungunya virus (CHIKV), a member of the genus *Alphavirus* in the family *Togaviridae*. The disease, which may manifest as febrile illness, is notorious for inflicting severe morbidity in form of prolonged joint pain which may persists for weeks or months in some patients [1]. Originally isolated in Tanzania in 1953 [2], the virus has spread causing major outbreaks in tropical Africa, islands in the Indian Ocean basin, and South-East Asia [3]. In November 2013, CHIKV was transmitted locally in the Americas for the first time, and over 2 million cases have been reported since then [4–6]. Similarly, locally transmitted cases have been detected in Europe [7,8]. In Kenya, major outbreaks occurred between 2004 and 2005 in Lamu and Mombasa in the Coastal Region, with at least 13,500 human cases, and as much as 75% of the population in Lamu affected [9]. In addition, a recent outbreak occurred in Mandera with 1,792 human cases recorded [10]. Overall, the ongoing expansion of its range presents a worrying public health trend at both local and global scales.

Both viral and vector factors have been ascribed to the global expansion of CHIKV. Amongst these is the rapid colonization and expanding habitat of the key *Aedes* species involved [11]. Interestingly, chikungunya outbreaks in West and Central Africa have tended to occur in smaller scales and largely in a sylvatic cycle involving humans and non-human primates and forest-dwelling *Aedes* species notably *Ae*. *furcifer-taylori group*, *Ae*. *africanus*, *Ae*. *luteocephalus*, and *Ae*. *neoafricanus* [12,13]. In stark contrast, larger scale outbreaks, mainly in urban and periurban settings, have largely been associated with the peridomestic and highly anthropophilic *Ae*. *aegypti*, as has been the case in recent outbreaks in East Africa, Asia, and the Americas [14,15].

Chikungunya has been reported in Mombasa city but so far has not been documented in Kisumu and Nairobi, although the presence and abundance of *Ae*. *aegypti* has been associated with urban areas [16,17]. Because the relative vector competence of different populations of *Ae*. *aegypti* can differ greatly for CHIKV [18], we hypothesized that the differences in the histories of chikungunya outbreaks in various areas in Kenya might be explained by the relative vector competence of these populations. Therefore, we tested populations of *Ae*. *aegypti* collected in Kilifi on the Coastal region near Mombasa, Nairobi, and Kisumu for their relative ability to transmit CHIKV.

## Methods

### Mosquito collection and rearing

*Aedes aegypti* was collected from selected sites in the three major cities in Kenya. Mosquitoes were collected as eggs using oviposition cups (black cups lined with oviposition papers and half filled with water) and as larvae from water holding containers in and around houses, between March and April 2016 (Table 1). The eggs and larvae were transported to the BSL-2 insectary at the Duduville Campus, International Centre of Insect Physiology and Ecology (ICIPE) in Nairobi, where the eggs were hatched and the larvae reared to provide F_0_ adult mosquitoes for this study. Adult mosquitoes were identified to confirm that they were *Ae*. *aegypti*, and a portion of them were blood fed on laboratory mice (Kenya Medical Research Institute, Animal House) to provide eggs. These were hatched, reared at 28°C and provided fish food (Tetramin) as larval food to produce F_1_ mosquitoes. The same procedure was used to produce F_2_ mosquitoes. Adult mosquitoes were provided 8% glucose as a carbohydrate source, which was replaced with water 24 hours prior to virus exposure. We used F_0-2_ mosquitoes in this study.

**Table 1:** *Aedes aegypti* strains tested for susceptibility to chikungunya virus.

Strain	Developmental stage collected	Location (Date of collection)
Mombasa	Larvae	Rabia-Kilifi County (March-April 2016)
Kisumu	Larvae	Kanyarkwar- Kisumu County (March 2016)
Nairobi	Eggs/Larvae	Githogoro-Nairobi County (March 2016)

### Virus amplification

The Lamu001 strain of an East/Central/South Africa lineage of CHIKV, isolated during the 2004–2005 outbreak on Lamu Island, was used for all the infection assays performed in this study. The virus was amplified in T-25 cell culture flasks (Corning Incorporated, USA) containing confluent monolayers of Vero cells (ATTC CCL-81), grown in cell culture media consisting of Minimum Essential Medium (MEM) (Sigma-Aldrich, St. Louis, MO) with Earle's salts and reduced NaHCO_3_, supplemented with 10% heat-inactivated fetal bovine serum (FBS) (Sigma-Aldrich), 2% L-glutamine (Sigma-Aldrich), and 2% antibiotic/antimycotic solution with 10,000 units penicillin, 10 mg streptomycin and 25μg amphotericin B per ml (Sigma-Aldrich,). The inoculated cells were incubated at 37°C for 1 hour, to allow for virus adsorption. Maintenance medium (MEM supplemented with 2% FBS) was then added, and the cells were incubated at 37°C in a 5% CO_2_ incubator and observed for cytopathic effects (CPE). After 24 hours, 80% CPE was observed and a portion of the CHIKV-media suspension cell suspension was harvested, added to defibrinated sheep blood (Central Veterinary Laboratories Kabete, Kenya) and used without freezing to produce an infectious blood meal used to expose mosquitoes to CHIKV.

### Vector competence

When the laboratory-reared, F_1_ or F_2_ mosquitoes from Mombasa, Kisumu, or Nairobi were 4–5 days old (or the F_0_ mosquitoes from Mombasa and Nairobi were 3–12 days old), they were exposed to blood meals containing four different titers. A Hemotek membrane feeding system (Discovery Workshops, Accrington, the United Kingdom), maintained at 37°C, was covered with mouse skin (Kenya Medical Research Institute, Animal House) and used for artificial blood feeding. All feeding was performed in a BSL-2 insectary at ICIPE.

In the first experiment, 100 μl of freshly harvested virus was added to 9.9 ml of cell culture media to produce a 1:100 stock virus. 3 ml of this stock was added to 7 ml of defibrinated sheep blood. We added 2 ml of the CHIKV-blood suspension to each well of a Hemotek feeder and *Ae*. *aegypti* from the three locations were allowed to feed for about 1 hour. Immediately after making the blood virus suspension, 100 μl of the suspension were added to 900 μL of homogenization media (MEM, supplemented with 15% FBS) to make a 1:10 suspension of the time-0 blood. At the end of the 1-hour feeding period, 100 μl of the virus blood meal were removed from one of the Hemotek feeders and added to 900 μl of homogenization media to determine an end of feeding concentration.

In a second experiment, conducted a few hours later, 3 ml of the freshly grown, CHIKV cell culture suspension were added directly to 7 ml of sheep blood to create a virus suspension. All other procedures remained the same as in the first experiment.

With all other procedures remaining the same, the experiments were repeated using a 1:10 dilution and undiluted freshly grown virus with titers different from those in the first two experiments. Therefore, in four separate experiments, mosquitoes were exposed to infectious blood meals containing different titers.

### Infection, dissemination and transmission assays

After feeding, all unengorged mosquitoes were removed and the cages containing the engorged mosquitoes were maintained in an insectary at 28°C, 12:12 (L:D) photoperiod, and a cotton pad containing 8% glucose solution was placed on top of the cage. On days 5, 7, 9, and 14, a sample of the mosquitoes was removed, placed in small plastic cups (covered with a fine netting material and secured with rubber bands), and cold anesthetized by placing in a refrigerator at -20°C for about 40 seconds.

The legs and wings of each mosquito were removed and the body placed on a sticky tape. The mosquito’s proboscis was inserted into a capillary tube containing 15–20 μl of homogenization media, and left to salivate for 30 minutes. The saliva containing media was eluted to 200 μl of homogenization media and the samples stored at -80°C until assayed for virus by cell culture. The body and legs were placed separately in microcentrifuge tubes containing 1 ml of homogenization media and stored at -80°C until assayed for virus by plaque assay.

### Virus and mosquito sample quantification

Quantification of CHIKV-blood meal, and mosquito body and leg samples was performed by plaque assay. Mosquito bodies were homogenized using a Minibeadbeater (BioSpec Products Inc, Bartlesville, OK 74005 USA) with the aid of a copper bead (BB-caliber airgun shot) and clarified by centrifugation at 12,000 rpm (Eppendorf centrifuge 5417R) for 10 mins at 4°C. Serial 10-fold dilutions were prepared and inoculated in 12-well plates containing confluent Vero cell monolayers. Each well was inoculated with 100 μl of virus/blood or body dilutions, incubated for 1 hour to allow for adsorption, with frequent agitation/rocking. The infected cells were then maintained using 2.5% methylcellulose mixed with 2X MEM and incubated at 37°C with 5% CO_2_. On the fourth day, the plates were fixed for 1 hour with 10% formalin, and then stained for 1 hour with 0.5% crystal violet solution. Plaques were counted on a light box. Only the legs of the positive mosquito bodies were homogenized and tested in the same way, to determine the dissemination rate.

To test for virus transmission, 80 μl of the saliva sample was inoculated into a well of a 24-well plate containing confluent Vero cell monolayers. Plates were incubated for 1 hour to allow for adsorption, with frequent agitation/rocking. The infected cells were maintained using maintenance media (1 ml per well) and incubated at 37°C with 5% CO_2_. Plates were observed for 7 days and the supernatant of wells showing CPE were harvested and virus quantified by plaque assay.

If the virus was detected in the mosquito’s body but not in the legs, the mosquito was considered to have a non-disseminated infection limited to the midgut. Detection of virus in the body and legs was considered as evidence of a disseminated infection [19]. All samples that contained CHIKV in their saliva were considered competent in transmitting the virus. The overall infection and dissemination rates for *Ae*. *aegypti* populations from Mombasa, Kisumu and Nairobi were compared using Chi-squared tests. Body titers for mosquitoes with disseminated and non-disseminated infections were compared using a t-test. All analysis was performed in R version 3.3.1 [20] at α = 0.05 level of significance. We used the exact (binomial) method of calculating 95% confidence intervals (C.I.) (https://measuringu.com/wald/).

### Ethical statement

Scientific and ethical approval was obtained from Kenya Medical Research Institute Scientific and Ethics Review Unit (KEMRI-SERU) (Project Number SERU 2787). The animal use component was reviewed and approved (approval number KEMRI/ACUC/ 03.03.14) by the KEMRI Animal Use and Care committee (KEMRI ACUC). The KEMRI ACUC adheres to national guidelines on the care and use of animals in research and education in Kenya enforced by National Commission for Science, Technology and Innovation (NACOSTI). The Institute has a foreign assurance identification number F16-00211 (A5879-01) from the Office of Laboratory Animal Welfare (OLAW) under the Public Health Service and commits to the International Guiding Principles for Biomedical Research Involving Animals.

## Results

### *Aedes aegypti* susceptibility to CHIKV infection

The titers in the infectious blood meals ranged from 105.6–10^7.5^ PFU/ml, with titers of pre- and post-feeding samples for each meal being nearly identical. Although the number of days post virus exposure at any given dose did not have any significant effect on susceptibility to the virus (Table 2), the overall infection rates for all three geographic populations increased with an increase in the exposure dose, with very low infection rates in all three populations when ≤10^5.9^ PFU/ml were ingested. At the higher exposure doses 10^6.9–7.5^, infection rates were highest in the mosquitoes derived from those collected in Mombasa. However, these differences were not statistically significant (χ2 ≤ 1.04, DF = 1, P ≥ 0.31).

**Table 2:** Infection rates by day after feeding on a chikungunya virus blood meal.

Strain	Generation	No. Infected/No. Tested (Infection rates) by days extrinsic incubation			
		5–7	9	14	Total^a^
Infectious blood meal = 10^5.6^ PFU/ml					
Mombasa	F_0_	0/2 (0)	n.t.	n.t.	0/2 (0, 0–78)
Kisumu	F_1_	0/10 (0)	n.t.	0/19 (0)	0/29 (0, 0–10)
Nairobi	F_0_	0/5 (0)	n.t.	1/6 (17)	1/11 (9, 0–41)
Infectious blood meal = 10^5.9^ PFU/ml					
Mombasa	F_2_	2/35 (6)	3/18 (17)	n.t.	5/53 (9, 3–21)
Kisumu	F_2_	8/40 (20)	1/20 (5)	1/18 (6)	10/78 (13, 8–25)
Nairobi	F_2_	2/30 (7)	1/10 (10)	n.t.	3/40 (8, 2–20)
Infectious blood meal = 10^6.9^ PFU/ml					
Mombasa	F_2_	16/26 (62)	n.t.	n.t.	16/26 (62, 40–80)
Kisumu	F_2_	16/40 (40)	10/20 (50)	10/16 (63)	36/76 (47, 36–59)
Nairobi	F_2_	15/30 (50)	4/7 (57)	n.t.	19/37 (51, 34–68)
Infectious blood meal = 10^7.5^ PFU/ml					
Mombasa	F_0_	8/8 (100)	n.t.	6/8 (75)	14/16 (88, 62–98)
Nairobi	F_0_	5/7 (71)	n.t.	8/9 (89)	13/16 (81, 54–96)

n.t., not tested (samples not collected on these days).

^*a*^Infection rate for all mosquitoes combined, number infected/number tested (infection rate, 95% confidence interval).

### *Aedes aegypti* susceptibility to CHIKV dissemination

In all three populations, viral dissemination was observed as early as 5–7 days of extrinsic incubation, when mosquitoes were exposed to titers of ≥10^5.9^ PFU/ml. Although dissemination rates appeared to increase with increasing exposure doses, virtually all of this increase could be accounted for by an increase in infection rates. Also, the observed dissemination rates at 10^6.9–7.5^ were highest in the population of mosquitoes from Mombasa but the difference was however not significant (χ^2^ = 3.66, DF = 2, P = 0.16). Regardless of mosquito origin, infectious dose, or period of extrinsic incubation, about 71% of the infected mosquitoes had developed a disseminated infection (Table 3).

**Table 3:** Dissemination rates by day after feeding on a chikungunya virus blood meal.

Strain	Generation	No. Disseminated/No. Tested (Dissemination rates) by days extrinsic incubation^a^				
		5–7	9	14	Total^b^	D_i_ Total^c^
Infectious blood meal = 10^5.6^ PFU/ml						
Mombasa	F_0_	0/2 (0)	n.t.	n.t.	0/2 (0, 0–78)	0 (0)
Kisumu	F_1_	0/10 (0)	n.t.	0/19 (0)	0/29 (0, 0–10)	0 (0)
Nairobi	F_0_	0/5 (0)	n.t.	1/6 (17)	1/11 (9, 0–41)	1/1 (100)
Infectious blood meal = 10^5.9^ PFU/ml						
Mombasa	F_2_	1/35 (3)	2/18 (11)	n.t.	3/53 (6, 1–16)	3/5 (60)
Kisumu	F_2_	5/40 (13)	1/20 (5)	1/18 (6)	7/78 (9, 4–18)	7/10 (70)
Nairobi	F_2_	1/30 (3)	0/10 (0)	n.t.	1/40 (3, 1–13)	1/3 (33)
Infectious blood meal = 10^6.9^ PFU/ml						
Mombasa	F_2_	12/26 (46)	n.t.	n.t.	12/26 (46, 27–67)	12/16 (75)
Kisumu	F_2_	11/40 (28)	5/20 (25)	4/16 (25)	20/76 (26, 17–38)	20/36 (56)
Nairobi	F_2_	9/30 (30)	4/7 (57)	n.t.	13/37 (35, 20–53)	13/19 (68)
Infectious blood meal = 10^7.5^ PFU/ml						
Mombasa	F_0_	8/8 (100)	n.t.	6/8 (75)	14/16 (88, 62–98)	14/14 (100)
Nairobi	F_0_	5/7 (63)	n.t.	8/9 (89)	13/16 (76 50–93)	13/13 (100)

n.t., not tested (samples not collected on these days).

^*a*^Dissemination rate = percentage of mosquitoes containing virus in their legs, regardless of infection status.

^*b*^Dissemination rate for all mosquitoes combined, number disseminated/number tested (dissemination rate, 95% confidence interval).

^*c*^Dissemination rate for all infected mosquitoes combined, number disseminated/number infected tested (dissemination rate for infected mosquitoes).

### *Aedes aegypti* ability to transmit CHIKV

Similar to viral dissemination, viral transmission was only detected in mosquitoes that ingested ≥10^5.9^ PFU/ml (Table 4). Although transmission rates appeared to increase with increasing exposure doses, virtually all of this increase was accounted for by an increase in dissemination rates. The transmission rates ranged from 0–2% at a viremia level of 10^5.9^, 3–15% at a viremia level of 10^6.9^, and 13–19% at a viremia level of 10^7.5^. Regardless of mosquito origin, infectious dose, or periods of extrinsic incubation, about 21% of the mosquitoes with a disseminated infection were able to transmit infectious virus to the capillary tube (Table 4).

**Table 4:** Transmission rates by day after feeding on a chikungunya virus blood meal.

Strain	Generation	No. Transmitted/No. Tested (Transmission rates) by days extrinsic incubation^a^				
		5–7	9	14	Total^b^	T.R.(D)^c^
Infectious blood meal = 10^5.6^ PFU/						
Mombasa	F_0_	0/2 (0)	n.t.	n.t.	0/2 (0)	0 (0)
Kisumu	F_1_	0/10 (0)	n.t.	0/19 (0)	0/29 (0)	0 (0)
Nairobi	F_0_	0/5 (0)	n.t.	0/6 (0)	0/11 (0)	0/1 (0)
Infectious blood meal = 10^5.9^ PFU/ml						
Mombasa	F_2_	0/35 (0)	1/18 (6)	n.t.	1/53 (2)	1/3 (33)
Kisumu	F_2_	1/40 (3)	0/20 (0)	0/18 (0)	1/78 (1)	1/7 (14)
Nairobi	F_2_	0/30 (0)	0/10 (0)	n.t.	0/40 (0)	0/1 (0)
Infectious blood meal = 10^6.9^ PFU/ml						
Mombasa	F_2_	4/26 (15)	n.t.	n.t.	4/26 (15)	4/12 (33)
Kisumu	F_2_	2/40 (5)	0/20 (0)	0/16 (0)	2/76 (3)	2/20 (10)
Nairobi	F_2_	1/30 (3)	4/7 (57)	n.t.	5/37 (14)	5/13 (38)
Infectious blood meal = 10^7.5^ PFU/ml						
Mombasa	F_1_	2/8 (25)	n.t.	1/8 (13)	3/16 (19)	3/14 (21)
Nairobi	F_0_	1/7 (13)	n.t.	1/9 (11)	2/16 (13)	2/13 (15)

n.t., not tested (samples not collected on these days).

^*a*^Transmission rate = percentage of mosquitoes from which virus was detected in the saliva, regardless of infection status.

^*b*^Transmission rate for all mosquitoes combined, number transmitting/number tested (transmission rate).

^*c*^Transmission rate for all mosquitoes with a disseminated infection combined, number transmitting/number disseminated (transmission rate for mosquitoes with a disseminated infection).

### Mosquito body and leg titers

Based on the titers detected for each population of *Ae*. *aegypti*, we observed that mosquitoes that were susceptible to infection and failed to disseminate the virus had titers at least a log lower than mosquitoes which were susceptible and had disseminated the virus (Table 5). This difference was significant (t = 8.10, DF = 4, P = 0.0012). In general, viral dissemination only occurred when body titers were ≥10^4^ in all populations (Table 5). However, for mosquitoes with a disseminated infection, no significant difference in leg titers was observed for those that did, or did not, transmit virus by bite (t = 0, DF = 4, P = 1.0).

**Table 5:** Mean body and leg titers for *Aedes aegypti* from three major cities in Kenya exposed to chikungunya virus.

Strain	Non disseminated	Disseminated^b^		Mean leg titer ^c^	
	Body titer^a^	Body Titer	Leg titer	N.T	Trans.
Mombasa	10^3.4^	10^4.8^	10^2.7^	10^2.6^	10^3.0^
Kisumu	10^3.4^	10^4.4^	10^2.6^	10^2.6^	10^2.6^
Nairobi	10^3.1^	10^4.5^	10^2.7^	10^2.8^	10^2.4^

^*a*^ Mean body titer for infected mosquitoes with negative legs (PFU/specimen).

^*b*^ Mean titers for infected mosquitoes with positive legs (PFU/specimen).

^*c*^ Mean leg titers for virus-positive legs with negative saliva (N.T. = nontransmitters) and those with positive saliva (Trans. = transmitters) (PFU/specimen).

## Discussion

Populations of *Ae*. *aegypti* from Mombasa, Nairobi, and Kisumu were all competent vectors of CHIKV. The recent outbreaks of chikungunya in Africa, the Americas, Asia, and Europe [7,10,21,22], clearly demonstrate the potential for CHIKV to spread to new geographical areas and cause massive epidemics. The most likely way that CHIKV may be introduced into new areas is by a person infected in an area where CHIKV is currently being transmitted traveling to an area where susceptible people and competent vectors exist. In addition, it is possible for an infected mosquito to be transported from an area of active transmission to an area with susceptible people and vectors [21,23]. The risk of importation of CHIKV into new areas is high because chikungunya epidemics often result in high attack rates, high viremia levels among infected individuals, as well as the widely distributed *Aedes* vectors [14]. Due to the movement of humans to cities, these areas may be associated with higher risk of vector-borne pathogens, such as CHIKV. *Aedes aegypti* remains the only known urban vector for CHIKV transmission in Kenya. The CHIKV titers used in this study to expose mosquitoes are similar to published viremia levels associated with human infections (often >10^5^ PFU/ml of blood) in nature [24].

All populations of *Ae*. *aegypti* tested were able to transmit CHIKV under laboratory conditions, indicating that mosquitoes in each of these areas were competent vectors of CHIKV. Although infection rates were higher among mosquitoes from Mombasa as compared to Kisumu and Nairobi, these differences were not statistically significant, suggesting a higher CHIKV susceptibility for the *Ae*. *aegypti* from Mombasa was not the primary reason for the increased risk of CHIKV transmission in this area. For all three *Ae*. *aegypti* populations, about 70% of mosquitoes that became infected with CHIKV developed a disseminated infection. Thus, a midgut infection barrier may be an important factor affecting vector competence, particularly at lower viremias, as was suggested in another study on the dengue virus [25]. In addition, only 18 (21%) of 84 mosquitoes with a disseminated infection transmitted CHIKV to a capillary tube indicating a significant salivary gland barrier, although a mosquito may secrete less virus into a capillary tube than it would when feeding on an animal [26]. Transmission rates are therefore often lower when they are determined by collection of saliva as compared to allowing the mosquito to feed on a susceptible animal [27]. Therefore, failure to detect CHIKV in the saliva collected in a capillary tube does not mean that the mosquito would not have transmitted the virus by bite if it fed on a susceptible human, and our transmission rates should be considered as minimum transmission rates.

Interestingly, for mosquitoes from each of the three sites, dissemination and transmission rates reached high levels by 5–7 days after virus exposure (Tables 3 and 4). Therefore, at the viremia doses and temperature (28°C) used in this study, *Ae*. *aegypti* would be able to attain peak transmission rates in less than 1 week after feeding on a viremic person. This extrinsic incubation period is shorter than those described for other viruses transmitted by *Ae*. *aegypti*, including 7 to 12 days at temperatures ≥30°C for DEN [28] and a median of 10 days at 25°C for yellow fever [29].

Although we observed a significant difference (P = 0.0012) in the body titers of mosquitoes that did, or did not, disseminate CHIKV, we did not observe any difference in leg titers for mosquitoes with a disseminated infection that did, or did not, transmit virus (P = 1.0). This suggests that the salivary gland barrier which determines the ability of the virus to penetrate into the salivary glands and be secreted into the saliva is independent of the body titer in a mosquito with a disseminated infection [30].

Although, transmission rates trended higher among the Mombasa populations, the differences were not statistically significant compared to the populations in Kisumu and Nairobi, or more importantly, biologically meaningful. The higher transmission rates observed in the mosquitoes from Mombasa are, however, consistent with the higher chikungunya epidemics in this part of the country. The lower temperatures in Nairobi (average monthly temperatures 22–28°C) as compared to those in Mombasa (average monthly temperatures 27°C—31°C) may be a major contributing factor to the absence of chikungunya in the Nairobi area, as earlier studies demonstrated that temperature plays a significant role in the susceptibility of *Ae*. *aegypti* to CHIKV [31,32]. However, temperature cannot explain the low infection rates with CHIKV in Kisumu as monthly temperatures there range from 28–30°C. Also, the *Ae*. *aegypti* populations in these urban areas of Kenya may differ in their blood feeding behavior. This may be because the subspecies present in Mombasa may be predominantly *Ae*. *aegypti aegypti*, which has been described as more anthropophagic than the more sylvatic *Ae*. *aegypti formosus* strain mostly found inland and in forests [33,34]. Increased feeding on humans would have a much larger effect on CHIKV transmission than a moderate difference in vector competence, and may partly explain why CHIKV remains essentially absent in Kisumu, despite its relatively high temperatures.

In conclusion, although all three populations of *Ae*. *aegypti* were competent laboratory vectors for CHIKV, the Mombasa population appeared to be slightly more competent than the population from Kisumu and Nairobi. Findings from this study clearly demonstrated the importance of viremia levels in *Ae*. *aegypti* susceptibility to CHIKV. Vector competence is an important prerequisite in evaluating risk of emergence of CHIKV in addition to vector densities and host preference evaluation. Surveillance and control of the domestic vector, *Ae*. *aegypti*, should remain the main focus in many disease control programs and should be performed routinely where the risk is found to be high.
